# Case report: Immune response characterization of a pseudoprogression in a PD-L1-negative, TMB-low, *KEAP1/STK11* co-mutated metastatic NSCLC

**DOI:** 10.3389/fimmu.2024.1437961

**Published:** 2024-08-07

**Authors:** Nicolas Roussot, Marion Thibaudin, Jean-David Fumet, Susy Daumoine, Léa Hampe, Cédric Rébé, Emeric Limagne, Aurélie Lagrange, Victor Herreros, Julie Lecuelle, Hugo Mananet, Alis Ilie, David Rageot, Romain Boidot, Vincent Goussot, Anthony Comte, Pierre Jacob, Françoise Beltjens, Anthony Bergeron, Céline Charon-Barra, Laurent Arnould, Valentin Derangère, Sylvain Ladoire, Caroline Truntzer, François Ghiringhelli

**Affiliations:** ^1^ Unité Formation Recherche (UFR) des Sciences de Santé, Université Bourgogne Franche-Comté, Dijon, France; ^2^ Cancer Biology Transfer Platform, Department of Biology and Pathology of Tumors, Georges-François Leclerc Anticancer Center, UNICANCER, Dijon, France; ^3^ Equipe Therapies and Immune Response in Cancers (TIRECS), Centre de Recherche INSERM LNC-UMR1231, Dijon, France; ^4^ Department of Medical Oncology, Centre Georges-François Leclerc, Dijon, France; ^5^ Department of Interventional Radiology, Centre Georges-François Leclerc, Dijon, France

**Keywords:** case report, NSCLC, pseudoprogression, chemoimmunotherapy, neoantigen, cold tumor, tumor neoantigen, metastatic NSCLC

## Abstract

A patient with a PD-L1-negative, TMB-low, *KEAP1/STK11* co-mutated metastatic non-small cell lung cancer (NSCLC) experienced a multisite radiological progression at 3 months after initiation of chemoimmunotherapy as first-line treatment for metastatic disease. After the radiological progression, while she was not undergoing treatment, the patient had spontaneous lesions shrinkage and further achieved a prolonged complete response. Genomic and transcriptomic data collected at baseline and at the time of pseudoprogression allowed us to biologically characterize this rare response pattern. We observed the presence of a tumor-specific T-cell response against tumor-specific neoantigens (TNAs). Endogenous retroviruses (ERVs) expression following chemoimmunotherapy was also observed, concurrent with biological features of an anti-viral-like innate immune response with type I IFN signaling and production of CXCR3-associated chemokines. This is the first biological characterization of a NSCLC pseudoprogression under chemoimmunotherapy followed by a prolonged complete response in a PD-L1-negative, TMB-low, *KEAP1/STK11* co-mutated NSCLC. These clinical and biological data underline that even patients with multiple factors of resistance to immune checkpoint inhibitors could trigger a tumor-specific immune response to tumor neoantigen, leading to complete eradication of the tumor and probably a vaccinal immune response.

## Graphical Abstract

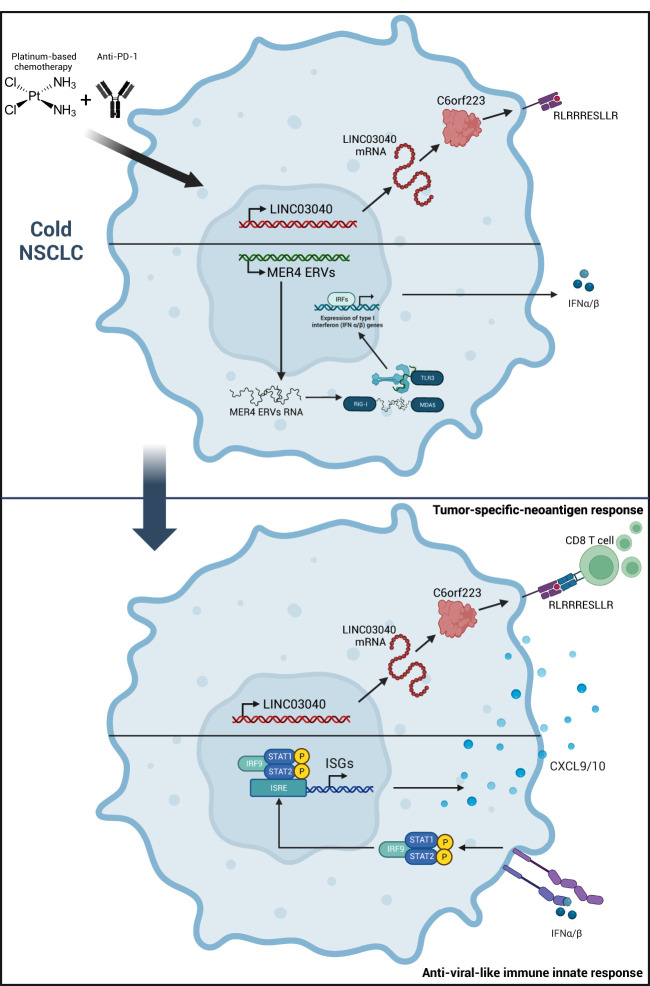

## Introduction

Pseudoprogression may be defined as a Response Criteria in Solid Tumors (RECIST)-defined response that occurred after RECIST-defined progression with a decrease in the sum of the longest diameter of the target lesions from the time of determination of disease progression rather than from baseline (1–3). In non-small cell lung cancer (NSCLC), pseudoprogression under immunotherapy monotherapy remains a rare event that does not exceed 7% (2). The artificial increase in tumor burden may be related to a transient immune-cell infiltrate in the tumor bed. Combining an immune checkpoint inhibitor (ICI) with cytotoxic chemotherapies may reduce the rate of pseudoprogression. Nevertheless, the biological insights that occurred behind this atypical pattern response have not been revealed.

This case presentation characterizes innate and adaptive immune mechanisms that arise in a patient with a PD-L1-negative, TMB-low (4), and *KEAP1/STK11* co-mutated metastatic NSCLC. These data demonstrate the appearance of a tumor-specific T-cell response against tumor-specific neoantigens (TNAs) with the stigma of an anti-viral immune response against endogenous retroviruses (ERVs), associated with a durable complete response.

## Results

### Case description

**Figure 1:**
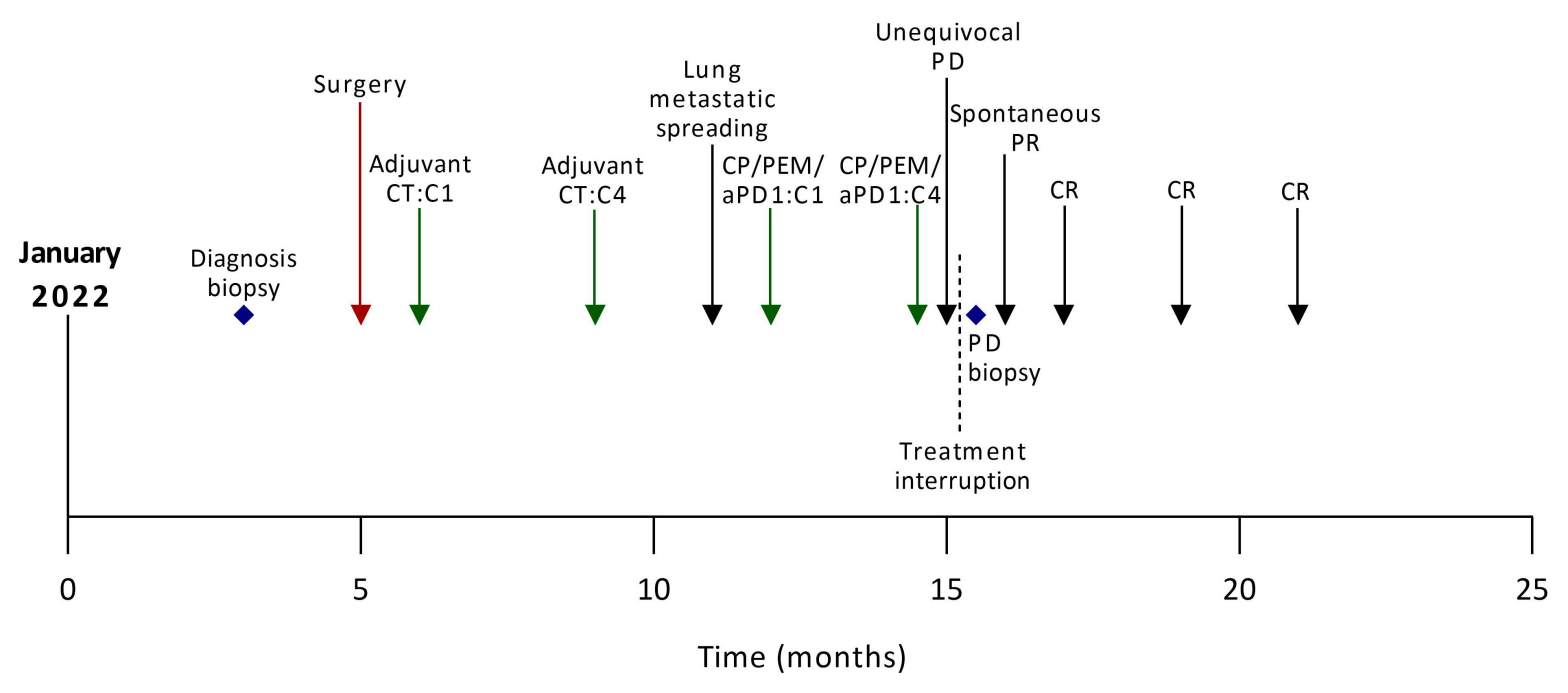
Case report timeline. aPD1, anti-Programmed cell Death 1; C, cycle; CP, carboplatin; CT, chemotherapy; CR, complete response, PD, progressive disease; PEM, pemetrexed; PR, partial response.

**Figure 2:**
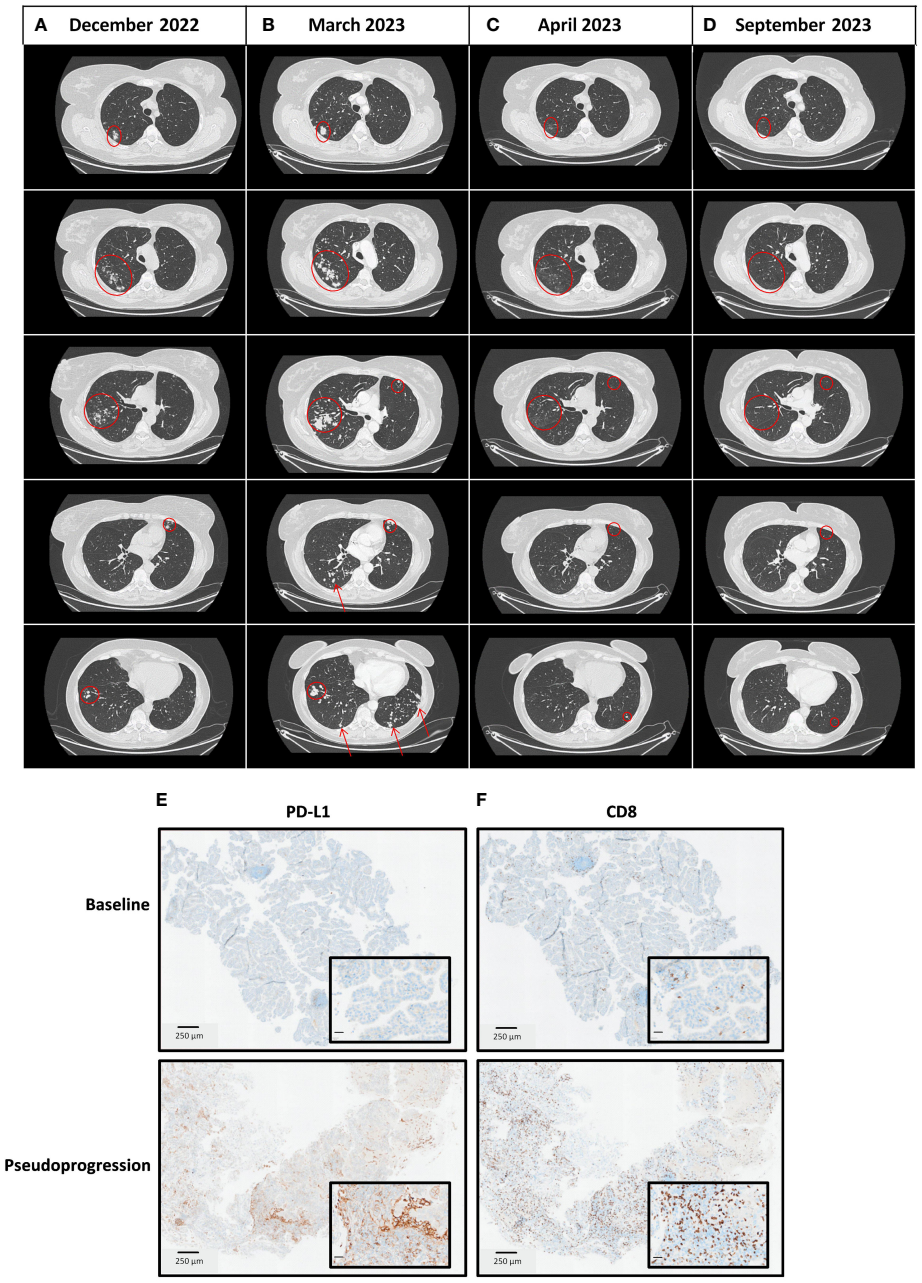
Thoracic CT scan imaging of the tumor response and immunohistochemistry analyses at different time points. **(A)** December 2022 at diagnosis of the metastatic disease with a metastatic spreading in both lungs. **(B)** March 2023 at the time of pseudoprogression after completion of 4 cycles of carboplatin-pemetrexed-pembrolizumab in the first-line setting of metastatic disease. **(C)** April 2023, four weeks after chemoimmunotherapy discontinuation, a thoracic CT scan was performed for radiotherapy preparation as part of the clinical trial in the second-line setting, in which the patient has been enrolled (NCT03431948). Lesions shrank spontaneously. **(D)** September 2023, six months after treatment discontinuation, a thoracic CT scan showed a prolonged complete response. **(E)** PD-L1 and CD8 staining on tumor biopsies at baseline (March 2022). **(F)** PD-L1 and CD8 staining on tumor biopsies at the time of pseudoprogression (March 2023).

A 54-year-old smoker woman has been diagnosed with lung adenocarcinoma of the lower left lobe, discovered in the context of a chronic cough, with no metastatic spreading (**Supplementary Figure 1**), in March 2022. The disease was negative for PD-L1 and CD8 staining. Whole exome sequencing (WES) identified a *KEAP1/STK11* co-mutation with loss of function for both genes, a *MAP2K1* activation, and an *ARID2* loss of function, as well as a low tumor mutational burden (TMB) with no actionable mutation (4.4 mut/Mb) (**Supplementary Table 1**). She underwent a lower left lobectomy with lymph node dissection that allowed the removal of an invasive lung adenocarcinoma with negative margins and no involved lymph nodes (0+/7); the disease stage was IIIA (pT4N0M0). The patient then received adjuvant chemotherapy comprising four cycles of cisplatin and navelbin which ended in September 2022. She remained disease-free until December 2022, when a computed-tomography (CT) scan identified an asymptomatic metastatic relapse with the apparition of multiple nodular lesions in both lungs, with no spreading out of the lungs (**Figures 1**, **2A–D**).

According to current guidelines, she received the first-line standard of care in this setting, which consisted of carboplatin, pemetrexed, and pembrolizumab. At the first tumor response evaluation after four cycles of chemoimmunotherapy in March 2023, while the patient has no performance status alteration, a CT scan showed a multisite progression with an unequivocal increase of target lesions (+ 68.7% according to the Response Evaluation Criteria in Solid Tumors, version 1.1), and non-target lesions associated with the apparition of new lesions in both lungs (**Figures 2A, B**; **Supplementary Table 2**). The treatment was stopped, a progressive lesion was biopsied, and it confirmed the presence of the patient’s lung adenocarcinoma. Consequently, she was screened for a clinical trial that combined immunotherapy and stereotactic body radiation therapy (SBRT) (NCT05259319) (5) in the second-line setting. The CT scan performed before patient enrollment showed that progressive lesions had spontaneously shrunk within a few weeks. Thus, the patient was not included in the study and received no further treatment. A complete response was later achieved and maintained while she was not undergoing treatment for 4 months (**Figures 2C, D**). In January 2024, the complete response was still ongoing.

### Analysis of the *in situ* immune response and genomic evolution before and after chemoimmunotherapy

On post-chemoimmunotherapy biopsies, the PD-L1 score has increased from 0 to 20%, along with a CD8 staining switch from moderate to intense (**Figures 2E, F**). RNAseq was used to analyze the immune context. MCP-counter (6) and Kassandra (7) software showed an enrichment of the adaptive immune cells after chemoimmunotherapy with increased expression of genes related to lymphocytes, T cells, CD8 T cells, CD4 T cells, and T helpers, but also with T regulators (Tregs) (**Supplementary Figures 2A, B**). We observed a decrease in TMB with no subclonal evolution (2.76 mut/Mb), suggesting that the tumor clone lost antigens following immune pressure (**Supplementary Table 1**). These findings suggest that the rapid, multisite, radiological progression was related to a pseudoprogression due to a massive infiltration of immune cells in lung metastatic lesions.

### Analysis of tumor-neoantigen-specific immune responses

**Figure 3:**
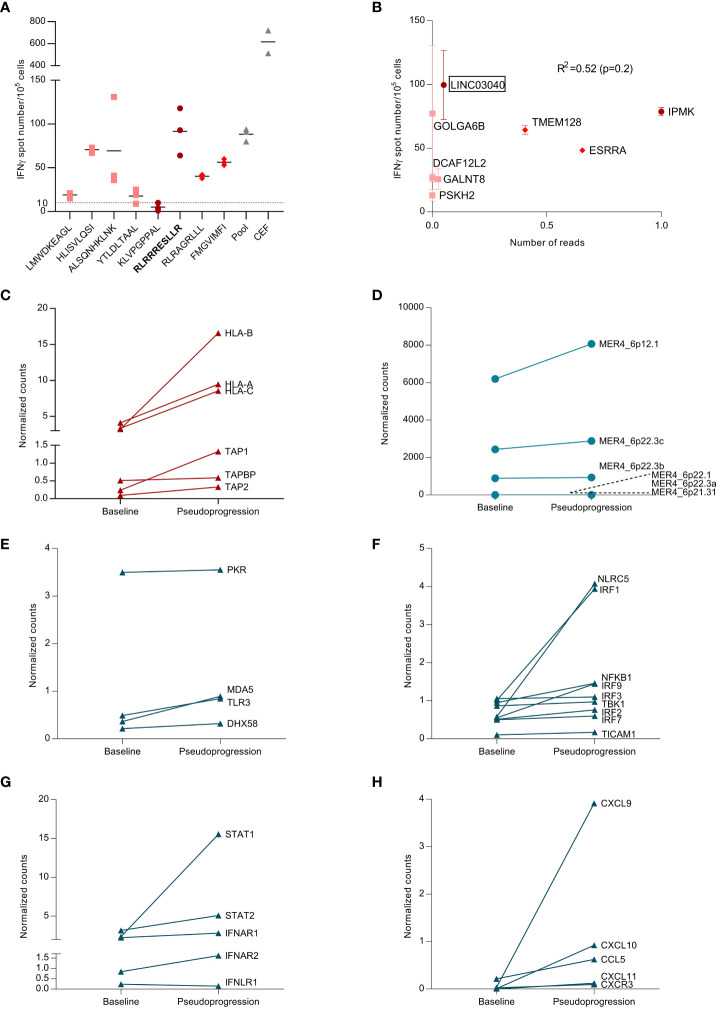
Analysis of tumor-neoantigen-specific immune response. **(A)** From a blood and tumor sample, exome sequencing was performed, and, using bioinformatics analysis, neopeptides found only in the tumor were identified. These peptides were synthesized, and the specific T response against these peptides was tested using blood and tumor samples to analyze the appearance of the specific anti-tumor response. Representative picture of *ex vivo* IFN-γ ELISpot using PBMCs takenat the time of pseudoprogression. Dot plot representing the number of IFN-γ spots for each condition (negative control, peptide pool, single peptide 1–8, and positive control (CEF pool)) in PBMCs at the time of pseudoprogression. Each number corresponds to the tested neoantigen. CEF, peptides from cytomegalovirus. Epstein–Barr virus and influenza virus, and pool corresponds to the pool of tested neoantigens. Dots represent technical replicates. Data are the mean ± s.d. **(B)** Correlation between the magnitude of the specific T response against neopeptides and the expression of genes coding for these neopeptides. Spearman correlation (R^2^=0.52, p-value=0.2). **(C)** Differential expression of adaptive immune response genes at baseline and at the pseudoprogression. Genes are expressed in counts, and counts were normalized with the ratio of initial counts divided by the median of household gene counts. **(D)** Differential expression of endogenous retroviruses (ERVs) between baseline and the pseudoprogression. Genes are expressed in counts, and counts were normalized with the ratio of initial counts divided by the median of household gene counts. **(E)** Differential expression of genes coding for double-stranded RNA (dsRNA) sensors between baseline and the pseudoprogression. Genes are expressed in counts, and counts were normalized with the ratio of initial counts divided by the median of household gene counts. **(F)** Differential expression of genes coding for proteins involved in RNA-sensing signaling between baseline and pseudoprogression. Genes are expressed in counts, and counts were normalized with the ratio of initial counts divided by the median of household gene counts. **(G)** Differential expression of genes coding for key regulators of type I IFN signaling between baseline and pseudoprogression. Genes are expressed in counts, and counts were normalized with the ratio of initial counts divided by the median of household gene counts. **(H)** Differential expression of genes coding for CCL5 and CXCR3-associated chemokines between baseline and pseudoprogression. Genes are expressed in counts, and counts were normalized with the ratio of initial counts divided by the median of household gene counts.

Based on the two WES analyses performed at diagnosis and at the time of pseudoprogression, the HLA class I neoantigen prediction pipeline [pVAC-Seq (8)] was applied to predict putative CD8-restricted neoantigens. Among these tumor-specific neoantigens, four were present only at baseline before surgery (LMWDKEAGL, HLISVLQSI, ALSQNHKLNK, YTLDLTAAL), two were present only after chemoimmunotherapy (RLRAGRLLL, FMGVIMFI), and two were found both at baseline and after chemoimmunotherapy (KLVPGPPAL, RLRRRESLLR) (**Supplementary Table 3**). The peptides were engineered, and an interferon (IFN)-γ ELISPOT assay was used to determine the magnitude of the neoantigen-specific T-cell response. There was heterogeneity in the magnitude of the response; the peptide that triggered the deepest T-cell response was RLRRRESLLR (**Figure 3A**). This peptide sequence belongs to the *LINC03040* gene (long intergenic non-protein-coding RNA 3040), located on chromosome 6 (6p21.1). In humans, *LINC03040* is more expressed in the lungs than in any other organ (9). Upon RNAseq, we observed a strong increase in expression of this gene in comparison to other genes related to neopeptides in the sample taken after chemoimmunotherapy (**Supplementary Figures 3A, B**). However, the number of neopeptide-derived gene counts after pseudogrogression was not correlated with the magnitude of response observed in ELISPOT (Spearman correlation, R^2^ = 0.52, p-value=0.2 **Figure 3B**). RNAseq analyses also revealed an expression increase of HLA class I (HLA-A, -B, and -C) transporters associated with antigen processing (TAP-1, TAP-2) and TAP-binding protein genes (**Figure 3C**). These findings confirmed that a tumor-neoantigen-specific T-cell response occurred at the time of pseudoprogression.

### Analysis of ERVs expression and innate immune response

Endogenous retroviruses (ERVs) are highly expressed in various cancer types and are associated with an increased innate immune response (10). We have demonstrated that high MER4 ERV expression predicts better outcomes in patients treated with immune checkpoint inhibitor (ICI) for NSCLC (11). We observed an increase in MER4 ERVs expression at the time of pseudoprogression. MER4_6p12.1 and MER4_6p22.3c were the most transcribed among several MER4 ERVs (**Figure 3D**). The HLA class I neoantigen prediction pipeline was applied to predict putative CD8-restricted neoantigens of these ERVs, but no ERV-specific antigen was detected. Nevertheless, RNAseq showed increased expression of double-stranded RNA (dsRNA) sensors such as TLR3, RIG-I and MDA5 (*IFIH1*) (**Figure 3E**). Genes coding for proteins involved in RNA-sensing signaling were also upregulated, particularly IRF1, IRF9, and NLRC5 (**Figure 3F**). The key regulators of type I IFNs signaling, STAT1 and STAT2, were strongly induced at the time of pseudoprogression (**Figure 3G**). Interferon-stimulated genes (ISGs) were further upregulated along with chemokine synthesis, particularly CCL5, CXCL9, and CXCL10. (**Figure 3H**; **Supplementary Figure 4**). Thus, we observed an increase in the expression of MER4 ERVs at the time of pseudoprogression, which was concurrent with biological features of an anti-viral-like innate immune response with type I IFNs signaling and CXCR3-associated chemokines release.

## Discussion

To our knowledge, we report the first case of a pseudoprogression with durable complete response that occurred spontaneously after an unequivocal progression under a standard chemoimmunotherapy regimen in a patient with a PD-L1-negative, TMB-low, *KEAP1/STK11* co-mutated NSCLC. The clinical presentation showed a rapid, multisite, unequivocal progression without performance status alteration at the first CT scan. The pseudoprogression was concomitant from a biological point of view as a switch from a cold to a hot tumor phenotype with CD8 T-cell recruitment. Moreover, we demonstrate a specific T-cell response against CD8-restricted neoantigens, associated with ERV expression and the biological features of an anti-viral-like innate immune response.

Although the treatment could have been pursued according to the iRECIST guidelines (12), given the very low rate of pseudoprogression observed under chemoimmunotherapy, the treatment was suspended. The combination of ICI with cytotoxic chemotherapies, which produce immediate tumor shrinkage, reduces the rate of pseudoprogression. In the Keynote-189 trial, 8.8% of patients had immediate progressive disease at the first tumor evaluation (13). Pathologic and molecular features also tend to make this patient a less suitable candidate for immunotherapy. Nevertheless, the first-line platinum, pemetrexed, and pembrolizumab combination improves survival in PD-L1-negative disease (14). Recent studies have investigated *KEAP1* and *STK11* mutations according to *KRAS* status and suggested that poor outcomes with immunotherapy are mostly found in the *KEAP1/KRAS* and *STK11/KRAS* co-mutated populations but not in the *KRAS* wild-type. *STK11* loss has been described as a predictive biomarker of early ICI failure when given as a monotherapy but not when combined with chemotherapy (15). The patient presented a *MAP2K1* mutation with possible activation. This is a rare (< 2% of NSCLC patients) (16–18) but detrimental mutation since it is associated with a shorter survival (18).

We identified eight CD8-restricted tumor-specific neoantigens present either at diagnosis, at the time of pseudoprogression, or both. The desperation of some TNAs concurrent with pseudoprogression suggests clearance of one or more tumor clones expressing these HLA class I neo-epitopes. TNAs may also be produced after chemotherapy exposition. We previously demonstrated that the platinum and pemetrexed combination is able to cause tumor immunogenic cell death (ICD) in a cold murine lung cancer model (19). Consistently, IHC analysis performed on progressive lesions under chemoimmunotherapy identified a massive increase in CD8 staining.

While MER4 ERVs expression has not led to an adaptive immune response since MER4 is not presented as an antigen by HLA class I, we observed stigmas of an antiviral-like innate immune response. RNAseq showed an enrichment of the antiviral immune signaling cascade, from RNA sensing to CXCR3-associated chemokines production. While ERVs expression is mostly silenced through epigenetic control in tissues, ERVs transcription is increased in several cancer types (10). Converging data suggest that ERV expression in tumors may promote an immune response, therefore increasing the ICI benefit (20, 21).

Together, these clinical and biological data underline that even patients with multiple factors of resistance to ICI could trigger a tumor-specific immune response to tumor neoantigens, leading to complete eradication of the tumor, and probably induce a vaccinal immune response against the tumor.

## Patient perspective

From the patient perspective, performance status was not altered at the time of pseudoprogression. Today, the patient has a good quality of life. The only sequellar toxicity is hypothyroidism, induced by immunotherapy and treated with thyroxine.

## Materials and methods

### Tumor response assessment

Tumor assessments were based on investigator-reported measurements and were performed according to RECIST version 1.1.

### PBMC isolation

After blood sampling in EDTA tubes, PBMCs were isolated from the whole blood by density gradient centrifugation (Lymphocyte Separation Medium, CMSMSL0101, Eurobio) with SepMate tubes (85460, STEMCELL Technologies). Whole blood was transferred into Sep-Mate tubes at a rate of 17 ml of whole blood per tube and then centrifuged at 1,200g for 10 min with an acceleration of 5 and the brake off. After removing as much plasma as possible, the phase containing the enriched PBMCs could be recovered. After washing with 45 ml of PBS, centrifugation of 300g for 7 min was carried out, and the PBMC pellet was resuspended in 5 ml of PBS 1× for counting. Then, a final wash with 10 ml of PBS 1× was performed before cryopreservation, which consisted of freezing at a rate of 8.10 (6) cells per cryotube in a solution of 50% FBS, 40% RPMI and 10% DMSO until further use.

### DNA and RNA extraction

After the evaluation of the tumor cell content in FFPE tumor specimens by a pathologist, samples were macro-dissected to obtain at least 80% tumor cell content for nucleic acid extraction. DNA was isolated from tumor tissue using the Maxwell 16 FFPE Plus LEV DNA Purification Kit (Promega). DNA from whole blood (germline DNA) was isolated using the Maxwell 16 Blood DNA Purification Kit (Promega) following the manufacturer’s instructions. The quantity of extracted genomic DNA was assessed by a fluorometric method with a Qubit device. RNA was extracted using the Maxwell RSC RNA FFPE Kit (Promega) according to the manufacturer’s protocol. DNA and RNA quality and quantity were assessed by spectrophotometry with absorbance at 230 nm, 260 nm and 280 nm. Tumor purity was reported in a table for each exome and RNA-seq data where information was available

### Whole-exome capture and sequencing

Two hundred nanograms of genomic DNA was used for library preparation, using the Agilent SureSelectXT Reagent Kit. The totality of the enriched library was used in the hybridization and captured with SureSelect All Exon v5 or v6 (Agilent) baits. After hybridization, the captured libraries were purified according to the manufacturer’s recommendations and amplified by polymerase chain reaction (12 cycles). Normalized libraries were pooled, and DNA was sequenced on an Illumina NextSeq 500 device using 2× 111-base pair (bp) paired-end reads and multiplexed.

### RNA-seq

RNA depleted of ribosomal RNA was used for the library preparation with a NEBNext Ultra II RNA Directional Library Prep Kit for Illumina according to the manufacturer’s instructions (New England Biolabs). RNA-seq was performed on a NextSeq 500 device (Illumina). The libraries were sequenced with 76-bp paired-end reads.

### Immunohistology procedure

Biopsies were collected before at baseline and at the time of Pseudoprogression, and were fixed after collection in paraformaldehyde and embedded in paraffin by the pathology laboratory. Four-micron slices were cut from FFPE tumor samples. The tissues embedded in paraffin were cut on a Leica rotary microtome (RM2145). For CD8 and PD-L1 staining, slides were deparaffinized and stained using a PT link (Agilent) and an Autostainer 48 (Agilent). In brief, slides were deparaffinized using a pH 9 buffer for 25 min at 95°C. After cooling, slides were washed in wash buffer (Agilent) twice for 5 min. Peroxydase blocking was performed with peroxydase blocking reagent (SM801, Agilent). Then, anti-human CD8 (1:100, clone C8/144B, M7103, Agilent) or anti-human PD-L1 (1/200, clone QR1, C-P0001–01, Diagomics) was added for 30 min at room temperature. EnVision FLEX HRP polymers (SM802, Agilent) were added for 15 min at room temperature after two washing steps. DAB (SM803, Agilent) was then added to samples for 2 min. After two new washing steps, slides were finally incubated with hematoxylin (SM806, Agilent) for 20 min and permanently mounted using a Leica automated coverslipper. Once stained and permanently mounted, slides were digitalized with NanoZoomer HT2.0 (Hamamatsu) at ×20 magnification to generate a whole slide imaging (WSI) file in.ndpi format.

### ELISpot assay

Circulating tumor-specific T cell responses were assessed by IFN-γ ELISpot after short-term *in vitro* stimulation of PBMCs with the eight engineered neopeptides. The eight neopeptides have been identified from patient exome analysis and expressed predominantly in the somatic exome (fold change (FC) in favor of tumor and median MT 50% inhibitory concentration (IC50) < 50). All synthetic peptides (>90% purity) were purchased from JPT Peptide Technologies GmbH. A mixture of peptides referred to as CEF, derived from influenza virus, Epstein–Barr virus and cytomegalovirus (Cellular Technology), was used to evaluate antiviral recall responses. In brief, the frozen PBMCs were thawed and cultured with tumor-derived peptides (5 μg ml−1). The culture was carried out in a 24-well plate (4 × 106 cells per well) in RPMI 5% human serum. IL-7 (5 ng ml−1, 200–07, PeproTech) and IL-2 (20 UI ml−1, 202-IL-010, Novartis) were added on days 1 and 3, respectively. On day 7 of cell culture, the presence of antigen-specific T cells was measured by IFN-γ ELISpot assay according to the manufacturer’s instructions. In brief, lymphocytes from *in vitro* stimulation (10^5^ per well) were incubated for 18 h at 37°C in an ELISpot plate pre-coated with anti-human IFN-γ monoclonal antibody, with or without peptide mixtures in X-VIVO 15 medium (BE04–418, Ozyme). Cells were cultured with medium alone and phorbol 12-myristate 13-acetate (1 ng ml−1, P8139, Sigma-Aldrich)/ionomycin (10 mmol L−1, I3909, Sigma-Aldrich) as negative and positive controls, respectively. The IFN-γ spots were revealed following the manufacturer’s instructions (Diaclone). The number of specific T cells expressed as ΔIFN-γ spots per 10^5^ cells was calculated after background value substraction (medium). Spot-forming cells were counted using the CTL Immunospot system (Cellular Technology). Responses were considered positive when the IFN-γ spots number was greater than 10 and greater than twice the background (22).

### Whole-exome sequencing data analysis

Reads in the FASTQ format were aligned to the reference human genome GRCh37 using the Burrows–Wheeler aligner (BWA version 0.7.17). Local realignment was performed using the Genome Analysis Toolkit (GATK version 4.1.3.0). Duplicate reads were removed using Picard version 2.5. In case of matched tumor-normal samples, somatic single-nucleotide variants (SNVs) were identified using a validated pipeline that integrated mutation calls from three different mutation callers. SNVs were called with VarScan (version 2.4.3) (23) and Mutect (version 1.1.7) (24) and insertion/deletions (indels) were called with VarScan and Strelka (version 2.9.2) (25)

TMB was calculated using the number of significant SNVs (with untranslated transcribed regions, synonyms, introns and intergenic SNVs filtered out) divided by the number of megabases covered at a defined level. To identify tumor-specific mutant peptides, pVAC-Seq (personalized Variant Antigens by Cancer Sequencing) was used (pVACtools version 1.5.4) (26). pVAC-Seq is based on HLA typing obtained by HLAminer (27). The MSI score was computed using MSIsensor (28). The HRD score was obtained through the scarHRD pipeline (29).

### RNA-seq data analysis

Raw FASTQ data were pseudo-aligned, and gene counts as well as transcripts per kilobase million (TPM) were quantified using Kallisto software (30). Kallisto transcript index used as reference was built from merged human cDNA and ncDNA files from the GRCh37 assembly Ensembl. Gene-level count and transcripts matrices were then created with the DESeq2 R package (31) (R-4.2.2, October, 2022). Low-count genes were pre-filtered by removing genes with too few reads. Based on the gene expression matrix of samples and gene sets of each signature of interest (KEGG pathways), signature scores were calculated using single-sample gene set enrichment analysis (ssGSEA) in the GSVA package (32). Tumor microenvironment (TME)-associated transcriptomic elements were quantified using MCP-counter and Kassandra, following respective guidelines. The MCP-counter method allows the robust quantification of the absolute abundance of eight immune and two stromal cell populations. Kassandra uses a tree machine learning algorithm for the deconvolution of cell proportions in tissue on different hierarchical levels.

For comparison of genes or ERV expression between baseline and pseudoprogression samples, raw counts were normalized by dividing them by the median of housekeeping gene counts.

## Data availability statement

The original contributions presented in the study are included in the article/**Supplementary Materials**, further inquiries can be directed to the corresponding author/s.

## Ethics statement

The studies involving humans were approved by Georges François Leclerc Cancer Center (Dijon, France) local ethics committee (13.085). The studies were conducted in accordance with the local legislation and institutional requirements. The participants provided their written informed consent to participate in this study. Written informed consent was obtained from the individual(s) for the publication of any potentially identifiable images or data included in this article.

## Author contributions

NR: Conceptualization, Data curation, Investigation, Methodology, Visualization, Writing – original draft. MT: Formal analysis, Investigation, Methodology, Visualization, Writing – review & editing. J-DF: Investigation, Supervision, Writing – review & editing. SD: Data curation, Formal analysis, Investigation, Writing – review & editing. LH: Data curation, Formal analysis, Investigation, Writing – review & editing. CR: Investigation, Supervision, Writing – review & editing. EL: Supervision, Writing – review & editing. AL: Investigation, Writing – review & editing. VH: Data curation, Investigation, Writing – review & editing. JL: Data curation, Investigation, Writing – review & editing. HM: Data curation, Formal analysis, Investigation, Writing – review & editing. AI: Data curation, Formal analysis, Investigation, Writing – review & editing. DR: Data curation, Formal analysis, Investigation, Writing – review & editing. RB: Data curation, Investigation, Resources, Writing – review & editing. VG: Data curation, Investigation, Resources, Writing – review & editing. AC: Data curation, Investigation, Resources, Writing – review & editing. PJ: Data curation, Investigation, Resources, Writing – review & editing. FB: Data curation, Investigation, Resources, Writing – review & editing. AB: Data curation, Investigation, Resources, Writing – review & editing. CC-B: Data curation, Investigation, Resources, Writing – review & editing. LA: Data curation, Investigation, Resources, Writing – review & editing. VD: Data curation, Investigation, Resources, Writing – review & editing. SL: Supervision, Writing – review & editing. CT: Formal analysis, Investigation, Methodology, Software, Supervision, Writing – review & editing. FG: Conceptualization, Investigation, Methodology, Supervision, Validation, Writing – review & editing.

## References

[1] Fujimoto D Yoshioka H Kataoka Y Morimoto T Hata T Kim YH . Pseudoprogression in previously treated patients with non–small cell lung cancer who received nivolumab monotherapy. J Thorac Oncol. (2019) 14:468–74. doi: 10.1016/j.jtho.2018.10.167 30468872

[2] Ferrara R Caramella C Besse B Champiat S . Pseudoprogression in non–small cell lung cancer upon immunotherapy: Few drops in the ocean? J Thorac Oncol. (2019) 14:328–31. doi: 10.1016/j.jtho.2018.12.011 30782378

[3] Borcoman E Kanjanapan Y Champiat S Kato S Servois V Kurzrock R . Novel patterns of response under immunotherapy. Ann Oncol. (2019) 30:385–96. doi: 10.1093/annonc/mdz003 30657859

[4] Greillier L Tomasini P Barlesi F . The clinical utility of tumor mutational burden in non-small cell lung cancer. Transl Lung Cancer Res. (2018) 7:639–46. doi: 10.21037/tlcr.2018.10.08 PMC6249623 30505708

[5] Roussot N Fumet J-D Limagne E Thibaudin M Hervieu A Hennequin A . A phase i study of the combination of atezolizumab, tiragolumab, and stereotactic body radiation therapy in patients with metastatic multiorgan cancer. BMC Cancer. (2023) 23:1080. doi: 10.1186/s12885-023-11534-6 37946136 PMC10633948

[6] Becht E De Reyniès A Giraldo NA Pilati C Buttard B Lacroix L . Immune and stromal classification of colorectal cancer is associated with molecular subtypes and relevant for precision immunotherapy. Clin Cancer Res. (2016) 22:4057–66. doi: 10.1158/1078-0432.CCR-15-2879 26994146

[7] Zaitsev A Chelushkin M Dyikanov D Cheremushkin I Shpak B Nomie K . Precise reconstruction of the TME using bulk RNA-seq and a machine learning algorithm trained on artificial transcriptomes. Cancer Cell. (2022) 40:879–894.e16. doi: 10.1016/j.ccell.2022.07.006 35944503

[8] Hundal J Carreno BM Petti AA Linette GP Griffith OL Mardis ER . pVAC-seq: A genome-guided in silico approach to identifying tumor neoantigens. Genome Med. (2016) 8:11. doi: 10.1186/s13073-016-0264-5 26825632 PMC4733280

[9] Duff MO Olson S Wei X Garrett SC Osman A Bolisetty M . Genome-wide identification of zero nucleotide recursive splicing in drosophila. Nature. (2015) 521:376–9. doi: 10.1038/nature14475 PMC4529404 25970244

[10] Rooney MS Shukla SA Wu CJ Getz G Hacohen N . Molecular and genetic properties of tumors associated with local immune cytolytic activity. Cell. (2015) 160:48–61. doi: 10.1016/j.cell.2014.12.033 25594174 PMC4856474

[11] Lecuelle J Favier L Fraisse C Lagrange A Kaderbhai C Boidot R . MER4 endogenous retrovirus correlated with better efficacy of anti-PD1/PD-L1 therapy in non-small cell lung cancer. J Immunother Cancer. (2022) 10:e004241. doi: 10.1136/jitc-2021-004241 35277462 PMC8919440

[12] Seymour L Bogaerts J Perrone A Ford R Schwartz LH Mandrekar S . iRECIST: guidelines for response criteria for use in trials testing immunotherapeutics. Lancet Oncol. (2017) 18:e143–52. doi: 10.1016/S1470-2045(17)30074-8 PMC5648544 28271869

[13] Gandhi L Rodríguez-Abreu D Gadgeel S Esteban E Felip E De Angelis F . Pembrolizumab plus chemotherapy in metastatic non–Small-Cell lung cancer. N Engl J Med. (2018) 378:2078–92. doi: 10.1056/NEJMoa1801005 29658856

[14] Garassino MC Gadgeel S Speranza G Felip E Esteban E Dómine M . Pembrolizumab plus pemetrexed and platinum in nonsquamous non–Small-Cell lung cancer: 5-year outcomes from the phase 3 KEYNOTE-189 study. JCO. (2023) 41:1992–8. doi: 10.1200/JCO.22.01989 PMC10082311 36809080

[15] Hong L Aminu M Li S Lu X Petranovic M Saad MB . Efficacy and clinicogenomic correlates of response to immune checkpoint inhibitors alone or with chemotherapy in non-small cell lung cancer. Nat Commun. (2023) 14:695. doi: 10.1038/s41467-023-36328-z 36755027 PMC9908867

[16] Wang W Xu C Wang D Zhu Y Zhuang W Fang M . P70.05 the association between MAP2K1 mutation class and clinical features in MAP2K1-mutant east asian non-small cell lung cancer patients. J Thorac Oncol. (2021) 16:S564. doi: 10.1016/j.jtho.2021.01.1016

[17] Cheng ML Lee JK Kumar R Klein H Raskina K Schrock AB . Response to MEK inhibitor therapy in *MAP2K1* ( *MEK1* ) K57N non–Small-Cell lung cancer and genomic landscape of MAP2K1 mutations in non–Small-Cell lung cancer. JCO Precis Oncol. (2022) e2200382. doi: 10.1200/PO.22.00382 36455195

[18] Smith MR Wang Y D’Agostino R Liu Y Ruiz J Lycan T . Prognostic mutational signatures of NSCLC patients treated with chemotherapy, immunotherapy and chemoimmunotherapy. NPJ Precis Onc. (2023) 7:34. doi: 10.1038/s41698-023-00373-0 PMC10042886 36973365

[19] Limagne E Nuttin L Thibaudin M Jacquin E Aucagne R Bon M . MEK inhibition overcomes chemoimmunotherapy resistance by inducing CXCL10 in cancer cells. Cancer Cell. (2022) 40:136–152.e12. doi: 10.1016/j.ccell.2021.12.009 35051357

[20] Chiappinelli KB Strissel PL Desrichard A Li H Henke C Akman B . Inhibiting DNA methylation causes an interferon response in cancer *via* dsRNA including endogenous retroviruses. Cell. (2015) 162:974–86. doi: 10.1016/j.cell.2015.07.011 PMC4556003 26317466

[21] Panda A De Cubas AA Stein M Riedlinger G Kra J Mayer T . Endogenous retrovirus expression is associated with response to immune checkpoint pathway in clear cell renal cell carcinoma. JCI Insight. (2018) 3:e121522. doi: 10.1172/jci.insight.121522 30135306 PMC6141170

[22] Moodie Z Price L Gouttefangeas C Mander A Janetzki S Löwer M . Response definition criteria for ELISPOT assays revisited. Cancer Immunol Immunother. (2010) 59:1489–501. doi: 10.1007/s00262-010-0875-4 PMC2909425 20549207

[23] Koboldt DC Zhang Q Larson DE Shen D McLellan MD Lin L . VarScan 2: Somatic mutation and copy number alteration discovery in cancer by exome sequencing. Genome Res. (2012) 22:568–76. doi: 10.1101/gr.129684.111 PMC3290792 22300766

[24] Benjamin D Sato T Cibulskis K Getz G Stewart C Lichtenstein L . Calling somatic SNVs and indels with Mutect2. [preprint]. Bioinf. (2019). doi: 10.1101/861054

[25] Kim S Scheffler K Halpern AL Bekritsky MA Noh E Källberg M . Strelka2: fast and accurate calling of germline and somatic variants. Nat Methods. (2018) 15:591–4. doi: 10.1038/s41592-018-0051-x 30013048

[26] Hundal J Kiwala S McMichael J Miller CA Xia H Wollam AT . pVACtools: A computational toolkit to identify and visualize cancer neoantigens. Cancer Immunol Res. (2020) 8:409–20. doi: 10.1158/2326-6066.CIR-19-0401 PMC7056579 31907209

[27] Warren RL Choe G Freeman DJ Castellarin M Munro S Moore R . Derivation of HLA types from shotgun sequence datasets. Genome Med. (2012) 4:95. doi: 10.1186/gm396 23228053 PMC3580435

[28] Middha S Zhang L Nafa K Jayakumaran G Wong D Kim HR . Reliable pan-cancer microsatellite instability assessment by using targeted next-generation sequencing data. JCO Precis Oncol. (2017), 1–17. doi: 10.1200/PO.17.00084 PMC6130812 30211344

[29] Sztupinszki Z Diossy M Krzystanek M Reiniger L Csabai I Favero F . Migrating the SNP array-based homologous recombination deficiency measures to next generation sequencing data of breast cancer. NPJ Breast Cancer. (2018) 4:16. doi: 10.1038/s41523-018-0066-6 29978035 PMC6028448

[30] Bray NL Pimentel H Melsted P Pachter L . Near-optimal probabilistic RNA-seq quantification. Nat Biotechnol. (2016) 34:525–7. doi: 10.1038/nbt.3519 27043002

[31] Love MI Huber W Anders S . Moderated estimation of fold change and dispersion for RNA-seq data with DESeq2. Genome Biol. (2014) 15:550. doi: 10.1186/s13059-014-0550-8 25516281 PMC4302049

[32] Hänzelmann S Castelo R Guinney J . GSVA: gene set variation analysis for microarray and RNA-seq data. BMC Bioinf. (2013) 14:7. doi: 10.1186/1471-2105-14-7 PMC3618321 23323831

