# Supplementary figures and images for: Case report: Immune response characterization of a pseudoprogression in a PD-L1-negative, TMB-low, KEAP1/STK11 co-mutated metastatic NSCLC
Source: Front Immunol. 2024 Aug 7;15:1437961. doi: 10.3389/fimmu.2024.1437961 (PMC11335479; doi:10.3389/fimmu.2024.1437961)

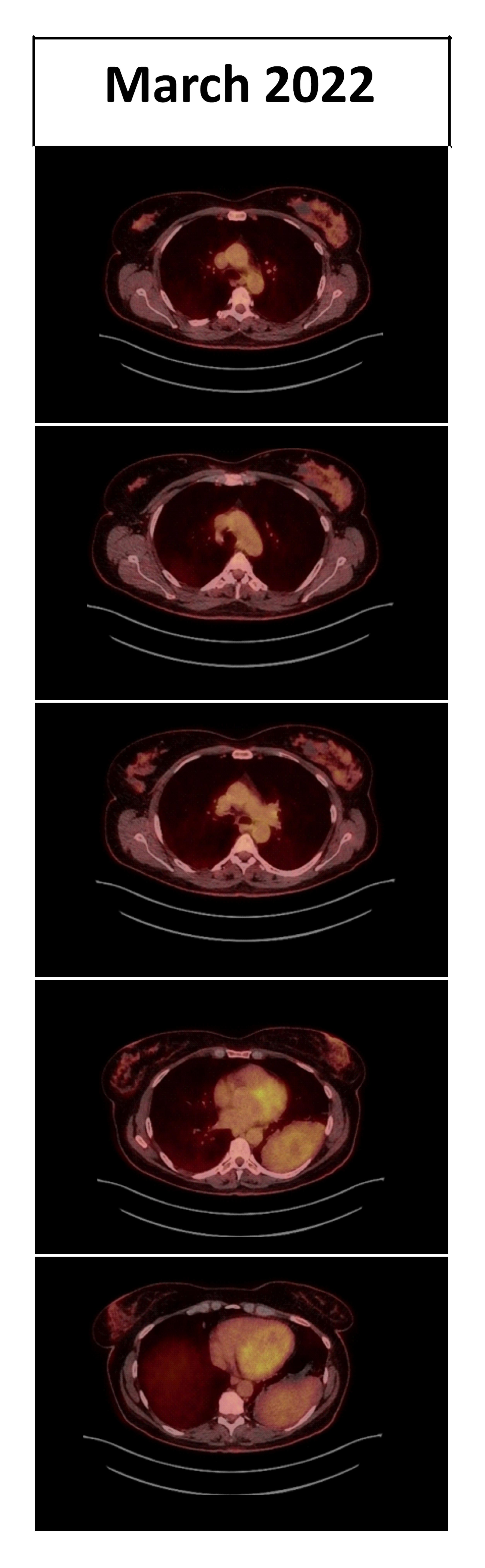

Supplement: Supplementary file 1 [file Image_1.jpeg]

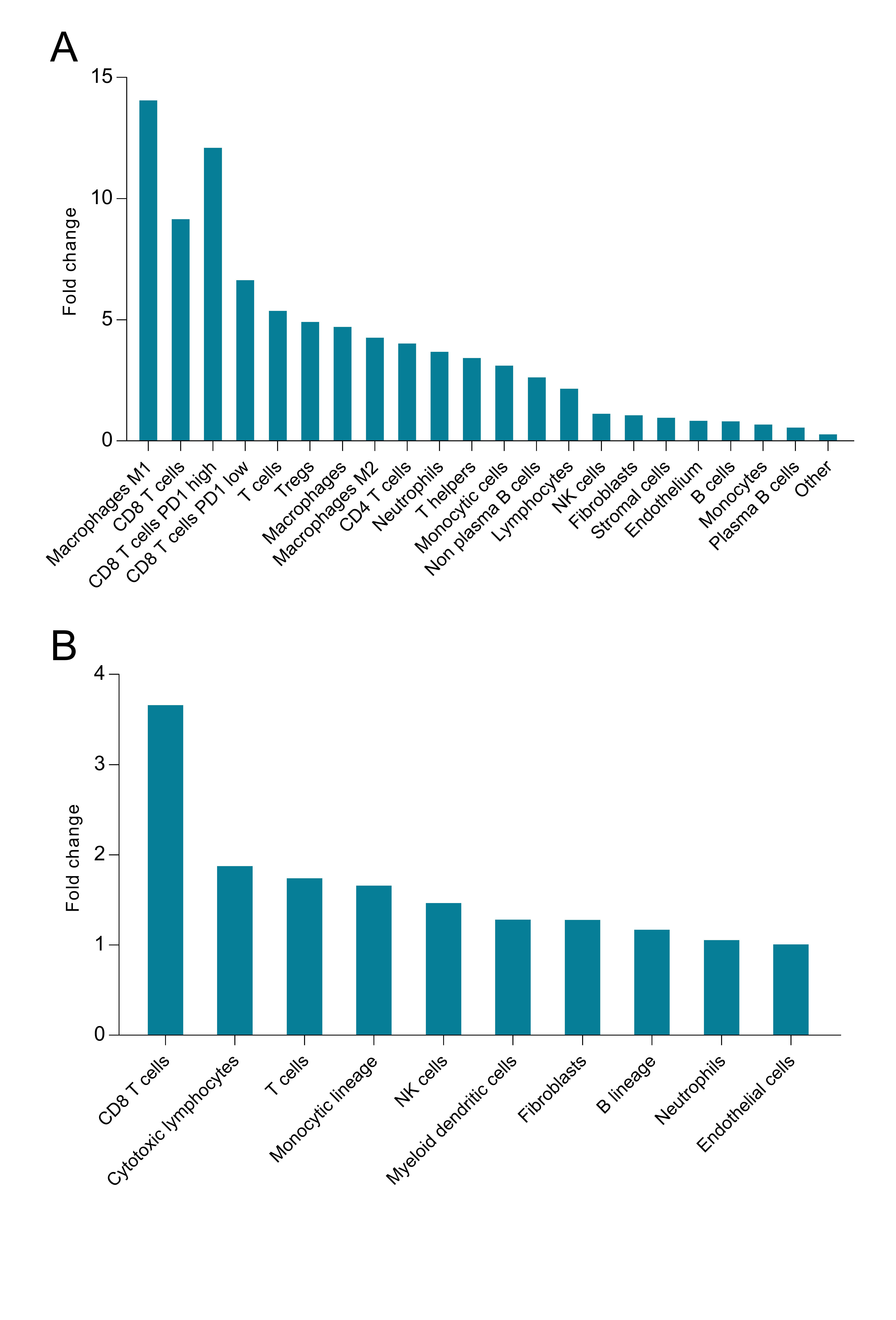

Supplement: Supplementary file 2 [file Image_2.jpeg]

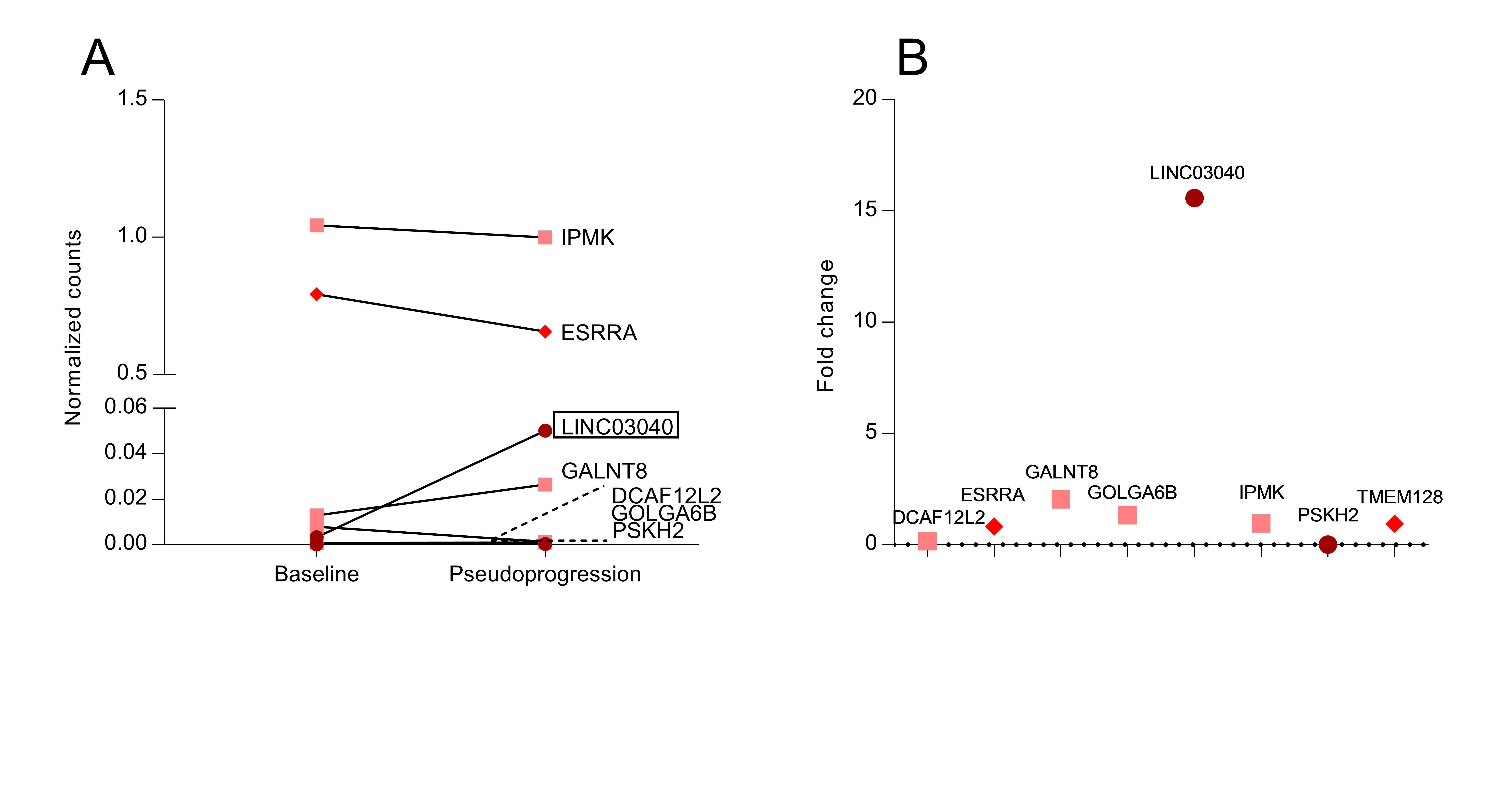

Supplement: Supplementary file 3 [file Image_3.jpeg]

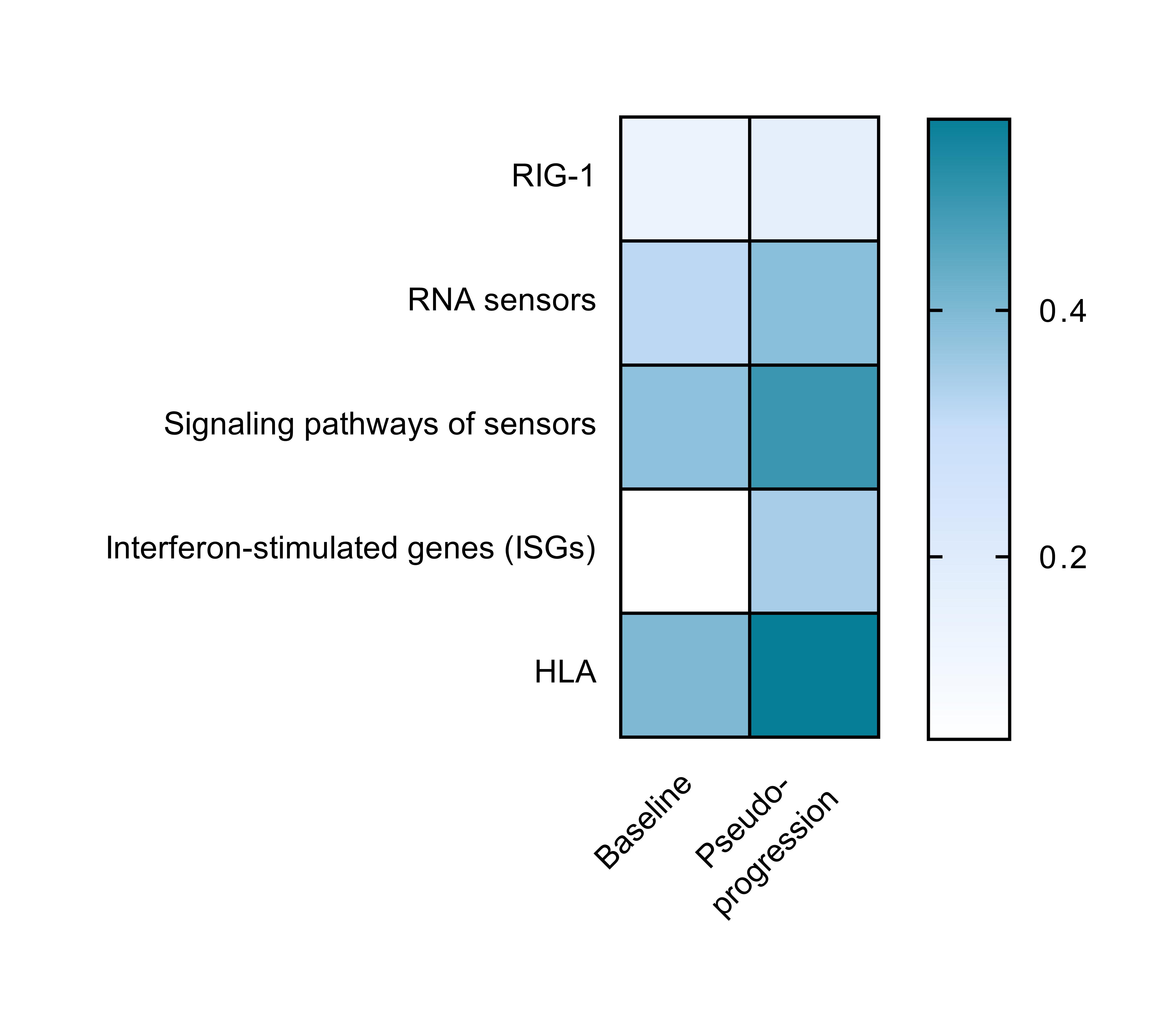

Supplement: Supplementary file 4 [file Image_4.jpeg]
