# Supplementary material for: Case report: Immune response characterization of a pseudoprogression in a PD-L1-negative, TMB-low, KEAP1/STK11 co-mutated metastatic NSCLC
Source: Front Immunol. 2024 Aug 7;15:1437961. doi: 10.3389/fimmu.2024.1437961 (PMC11335479; doi:10.3389/fimmu.2024.1437961)
Supplement: Supplementary file 5 [file Table_1.docx]

| **March 2022** | | | | | | **March 2023** | | | | | |
| --- | --- | --- | --- | --- | --- | --- | --- | --- | --- | --- | --- |
| PD-L1 score | | CD8+ staining | | | | PD-L1 score | | CD8+ staining | | | |
| Negative | 0% | Positive | | Moderate | | Positive | 20% | Positive | | Intense | |
| Gene | Nucleotidic variation | Proteic variation | | Status | Implications | Gene | Nucleotidic variation | Proteic variation | | Status | Implications |
| *ARID2* | c.712A>T | p.Lys238Ter | | Somatic | Loss of function | *ARID2* | c.3109C>T | p.Gln1037Ter | | Somatic | Loss of function |
| *ARID2* | c.3109C>T | p.Gln1037Ter | | Somatic | Loss of function |  |  |  | |  |  |
| *MAP2K1* | c.313_318del | p.Pro105_Ala106del | | Somatic | Possible activation |  |  |  | |  |  |
| *STK11* | c.581A>T | p.Asp194Val | | Somatic | Possible loss of function |  |  |  | |  |  |
| *KEAP1* | c.328A>G | p.Met110Val | | Somatic | Possible loss of function | KEAP1 | c.328A>G | p.Met110Val | | Somatic | Possible loss of function |
| TMB score | | | Status | | | TMB score | | | Status | | |
| 4.42 mut/Mb | | | TMB-low | | | 2.76 mut/Mb | | | TMB-low | | |
| MSI score | | | Status | | | MSI score | | | Status | | |
| 0.64 | | | MSS | | | 0.28 | | | MSS | | |
| HRD score | | | Status | | | HRD score | | | Status | | |
| 15 | | | HRP | | | 19 | | | HRP | | |
| Number of TCR clones | | | | | | Number of TCR clones | | | | | |
| 28 | | | | | | 26 | | | | | |

**Supplemental Table 1**. **Pathologic and molecular characteristics of the patient’s tumor at baseline (March 2022) and at the time of pseudoprogression (March 2023)**.
See point Materials and Methods for Whole Exome Sequencing data analysis. Both samples were biopsies performed by interventional radiology. The same operator performed biopsies each time.

|  | **December 2022** | **March 2023** |
| --- | --- | --- |
| **Lesions** | Date of metastatic spreading | Date of pseudoprogression |
| **Target lesions** | | |
| **Right upper lung 1** | 11.4 mm | 20.1 mm |
| **Right upper lung 2** | 16.4 mm | 26.8 mm |
| **Sum** | 27.8 mm | 46.9 mm |
| **Response** | X | Progression (+68.7%) |
| **Non-target lesions** | | |
| **Right lower lung 1** | 7.2 mm | 10.3 mm |
| **Right lower lung 2** | 6.5 mm | 10.7 mm |
| **Sum** | 13.7 mm | 21 mm |
| **Response** | X | Unequivocal progression (+34.7%) |
| **New lesions** | | |
| **Left lung 1** | X | 8.9 mm |
| **Left lung 2** | X | 11.5 mm |
| **Sum** | X | 20.8 mm |
| **New lesions** | X | >2 |
| **Response** | X | Progression |
| **Overall response** | | |
| **Overall response** | X | Progression |

**Supplemental Table 2**. Table summarizing tumor response assessment after completion of 4 cycles of carboplatin-pemetrexed-pembrolizumab in the first-line setting of metastatic disease. The RECIST v1.1 classification was used to create a baseline in December 2022 and to assess tumor response at each evaluation.

| Sequence | Gene name | Time |
| --- | --- | --- |
| LMWDKEAGL | DCAF12L2 | Baseline □ |
| HLISVLQSI | IPMK | Baseline □ |
| ALSQNHKLNK | GOLGA6B | Baseline □ |
| YTLDLTAAL | GALNT8 | Baseline □ |
| KLVPGPPAL | PSKH2 | Baseline/Pseudoprogression ○ |
| **RLRRRESLLR** | **LINC03040** | Baseline/Pseudoprogression ○ |
| RLRAGRLLL | ESRRA | Pseudoprogression ◊ |
| FMGVIMFI | TMEM128 | Pseudoprogression ◊ |

**Supplemental Table 3**. Putative CD8-restricted neoantigens determined by the HLA class I neoantigen prediction pipeline along with corresponding genes. The right column indicated the timepoint at which these neopeptides were presented. The pink square represents a neopeptide found at baseline, the dark red circle represents a neopeptide found at baseline and at pseudoprogression, and the red diamoind represents a neopeptide found at pseudoprogression.
